# Immunoinformatics-driven design of a multi-epitope vaccine against Seoul Virus: Structural, dynamic, and immunogenic profiling

**DOI:** 10.1016/j.clinsp.2026.100991

**Published:** 2026-05-12

**Authors:** Muhammad Naveed, Muhammad Asim, Tariq Aziz, Hafiz Muzzammel Rehman, Wafa Abdullah I. Al-Megrin, Ashwag Shami, Maher S. Alwethaynani, Abdullah A. Alqasem, Ahmad A. Alghamdi, Mohammed F. Abuzinadah, Ahmed M. Abdulfattah, Majid Alhomrani

**Affiliations:** a Department of Biotechnology, Faculty of Science and Technology, University of Central Punjab, Lahore, Pakistan; b Laboratory of Animal Health, Hygiene and Food Quality University of Ioannina Arta Greece; c School of Biochemistry & Biotechnology, University of the Punjab, Lahore, Pakistan; d Department of Biology, College of Science, Princess Nourah bint Abdulrahman University, P.O. Box 84428, Riyadh 11671, Saudi Arabia; e Department of Clinical Laboratory Sciences, College of Applied Medical Sciences, Shaqra University, Alquwayiyah, Riyadh, Saudi Arabia; f Department of Medical Laboratory, College of Applied Medical Sciences, Prince Sattam bin Abdulaziz University, Saudi Arabia; g Department of Clinical Laboratory Sciences, College of Applied Medical Sciences, Taif University, Saudi Arabia; h Department of Medical Laboratory Sciences, Faculty of Applied Medical Sciences, King Abdulaziz University, Saudi Arabia

**Keywords:** Seoul virus, Hemorrhagic fever with renal syndrome (HFRS), Multi-epitope vaccine, Molecular Docking, Molecular Dynamics Simulations

## Abstract

**Background and objective:**

Seoul Virus (SEOV), a zoonotic hantavirus, is a major cause of Hemorrhagic Fever with Renal Syndrome (HFRS) and represents a global health challenge. With no licensed vaccines or specific antivirals available, this study aimed to design a glycoprotein-based multi-epitope vaccine against SEOV using immunoinformatics approaches.

**Material*s* and methods:**

MHC-I and MHC-II epitopes were predicted via the Immune Epitope Database (IEDB) and screened for antigenicity, non-allergenicity, and non-toxicity. Four constructs were designed, with V2 selected as the best candidate. Structural modeling was performed using AlphaFold3 and validated with Ramachandran analysis. Molecular docking with Toll-like receptor-4, molecular dynamics simulations, and immune simulations were conducted to evaluate stability and immunogenicity.

**Results:**

The V2 construct demonstrated high antigenicity (0.683) and broad population coverage (95.94%). Structural validation confirmed 95.6% of residues in favored regions. Docking revealed strong binding to Toll-like receptor-4, and dynamics confirmed stability through RMSD, RMSF, Rg, PCA, DCCM, and FEL analysis. Immune simulations predicted robust responses, including high IgG1 titers. Codon optimization (GC content 56.62%) and cloning confirmed expression potential.

**Conclusions:**

The V2 construct exhibits stability, strong immunogenicity, and broad population coverage, supporting its potential as a vaccine candidate against SEOV. Experimental validation *in vitro* and *in vivo* is required to confirm efficacy and safety.

## Highlights

• Immunoinformatics-based design of a multi-epitope vaccine against Seoul virus (SEOV).
• Designed Vaccine construct V2 showed 95.94% global coverage and strong antigenicity.
• Docking and molecular dynamics simulations confirmed stable binding of V2 with the TLR4 receptor.
• Immune simulations predicted strong IgG1, IFN-γ, and memory responses of V2.
• Codon optimization and *in silico* cloning confirmed expression feasibility.

## Introduction

The zoonotic pathogen Seoul virus (SEOV), a member of the *Hantavirus* genus, poses a significant public health threat.^1^ It is primarily transmitted to humans through exposure to the urine, saliva, or feces of infected rodents, specifically *Rattus norvegicus* (Norway rats) and *Rattus rattus* (black rats).^2^ SEOV is a known cause of Hemorrhagic Fever with Renal Syndrome (HFRS), a potentially severe illness characterized by fever, bleeding, and kidney dysfunction. Since its initial discovery in Seoul, South Korea, the virus has spread widely due to the global migration of its rodent hosts.^3^ The immune response to SEOV involves both humoral and cellular components. Initially, IgM antibodies are produced during the acute phase and play a crucial role in the early neutralization of the virus.^4^ This is later followed by the production of IgG antibodies that maintain protection over time. CD8⁺ cytotoxic T lymphocytes are pivotal in the cell-mediated immune response to SEOV by eliminating virus-infected cells through MHC class I-restricted antigen recognition.^5^ CD4⁺ T-cells, activated through MHC class II pathways, act as helper T-cells that stimulate B-cells for class-switching and production of specific antibodies, in addition to secreting antiviral cytokines IFN-γ and IL-2.^6^ The coordination of these responses is essential for effective viral clearance and the establishment of long-term immunity. The presence of SEOV in both rural and urban areas, characterized by high levels of human and rodent interaction, enables it to spread globally. Reports of infection with the Seoul virus have been documented in many parts of the world, including Asia, Europe, and North America.^7^ In China and South Korea, SEOV is responsible for HFRS, with thousands of documented cases reported every year.^8^ In the United States, SEOV outbreaks have been linked to domestic rat breeding facilities, with a notable 2017 epidemic involving several human cases traced to pet rat exposure.^9^

Currently, no specific antiviral medications are approved for the treatment of SEOV-induced Hemorrhagic Fever with Renal Syndrome. Supportive therapy remains the primary approach, focusing on fluid management and dialysis in cases of severe kidney failure.^10^ The antiviral drug ribavirin is being used to treat hantavirus infections; however, it has a limited scope of effectiveness and high side effects. Additionally, the lack of a licensed vaccine targeting SEOV remains one of the significant shortcomings in controlling HFRS disease.^11^ Furthermore, the absence of specific therapeutic measures underscores the need to develop more effective strategies aimed at reducing SEOV transmission and reducing its public health impact.

The implementation of immunoinformatics has revolutionized vaccine development through the rapid, economical, and precise detection of immunogenic epitopes. This approach applies bioinformatics alongside immunological information to predict and evaluate B-cell, T-cell, and helper T-cell epitopes, verifying their requirements for inclusion in a vaccine.^12^ Immunoinformatics has already demonstrated significant progress in developing vaccine candidates for a wide range of infectious diseases, including SARS-CoV-2.^13^ Through the use of immunoinformatics, vaccine development is simplified by minimizing the classical trial-and-error processes, supporting time and cost efficiency, resource conservation, and enhancing the reliability of vaccine precision.^14^

The objective of this study is to design a multiepitope vaccine against the Seoul virus using immunoinformatic approaches. By identifying highly immunogenic MHC-I and MHC-II epitopes, the study aims to develop a vaccine candidate that elicits a robust and protective immune response. The design of the proposed vaccine candidate seeks to induce a strong immune response by incorporating potent T-cell epitopes that demonstrate high immunogenicity. Such methods present a paradigm shift in efforts to develop an SEOV vaccine that is safe and effective, as they are less costly and time-efficient than traditional methods, thereby overcoming the existing barrier in preventive measures.

## Materials and methods

### Protein retrieval

The Envelopment glycoprotein, with a UniProt ID: P28729, of the Seoul virus was selected as the target antigen due to its immune system domain tagging and its role during viral entry. The protein sequence was retrieved from the UniProt database (https://www.uniprot.org/), one of the most popular databases for protein sequences and functional data.^15^ For topology mapping of the glycoprotein, the TMHMM server (https://services.healthtech.dtu.dk/services/TMHMM-2.0/) was used.^16^

### Phylogenetic analysis

To assess the evolutionary relationship of the envelope glycoprotein selected for vaccine design against the Seoul virus, a phylogenetic analysis was conducted. The relevant protein sequence was processed through the BLASTp algorithm to find corresponding sequences within the relevant virus family. The received sequences were aligned, and a phylogenetic tree was generated using MEGA11 through the Neighbor-Joining (NJ) approach. A bootstrap analysis was conducted with 1000 replicates to test the confidence levels of the obtained tree branches. This approach illustrates how the selected protein is evolutionarily conserved, thus confirming that there is minimal change in the structure of the differentiated proteins across various species, making it an optimal target for vaccine development.^17^

### *In silico* prediction of T-cell epitopes

The Immune Epitope Database (IEDB) was utilized for predicting T-cell epitopes. Cytotoxic T-Lymphocyte (CTL) epitopes (MHC class I) were identified using the IEDB ANN 4.0 prediction server (http://tools.iedb.org/main/tcell/), while helper T-lymphocyte epitopes (MHC class II) were predicted using the IEDB NN-align 2.3 (NetMHCIIpan 2.3) server (http://tools.iedb.org/mhcii/). For both MHC-I and MHC-II, only epitopes with a predicted binding affinity of IC₅₀ < 100 nM were retained for downstream analysis. The selected epitopes were then evaluated for allergenicity and antigenicity to assess their immunogenic potential while ensuring safety parameters.^18^

### Epitopes evaluation

The predicted epitopes were first filtered based on their binding affinity, and only those with strong predicted interaction with MHC molecules (IC₅₀ < 100 nM) were retained for further analysis. These epitopes were then evaluated for antigenicity using VaxiJen v2.0 (https://www.ddg-pharmfac.net/vaxijen/VaxiJen/VaxiJen.html), and only highly antigenic epitopes with VaxiJen scores > 0.7 were considered, to ensure a strong potential to provoke an immune response. Allergenicity was assessed using AllerTOP v2.1 (https://www.ddg-pharmfac.net/allertop_test/), confirming that the selected epitopes were non-allergenic and therefore safe for humans. Toxicity analysis was performed using ToxinPred (https://webs.iiitd.edu.in/raghava/toxinpred/), and only non-toxic peptides were retained. In addition, priority was given to epitopes that targeted the greatest number of high-frequency HLA alleles, and highly overlapping or redundant epitopes that did not increase cumulative HLA allele coverage or representation of distinct regions of the target protein were removed.^19^ Accordingly, the final selection of 16 MHC-I and 16 MHC-II epitopes represents the outcome of a rigorous, multi-tiered refinement process, rather than a numerically arbitrary or methodologically unsupported decision.

### Population coverage analysis

To ensure vaccine efficacy across diverse human populations, population coverage analysis was performed using the IEDB Population Coverage tool (http://tools.iedb.org/population/). This analysis estimates, for each geographic region, the proportion of individuals predicted to present at least one of the selected epitopes on their HLA molecules, based on known HLA allele frequency data. In this way, the tool helps identify epitope sets that are collectively recognized by multiple HLA alleles and are therefore likely to provide broad global and regional coverage.^20^

### Vaccine construct design and evaluation

The vaccine construct design was achieved by choosing MHC-I and MHC-II epitopes with high antigenicity, non-allergenic, and non-toxic properties. For improved structural organization and antigen presentation, GPGPG linkers were used between MHC-II epitopes, while AAY linkers were placed between MHC-I epitopes to facilitate better cytotoxic T-lymphocyte processing. Furthermore, to enhance immune responses, β-defensin-3 adjuvant was included at the C-terminus. Additionally, a PADRE sequence was added to the N-terminal to enhance T-cell activation, and a 6X histidine tag was included for purification during expression studies. To improve vaccine efficacy, four different vaccine constructs (V1, V2, V3, and V4) were constructed, each with a different combination of epitopes to determine the optimal configuration. The constructs are evaluated for antigenicity using VaxiJen v2.0, allergenicity with AllerTOP v2.0, and toxicity using ToxinPred.^21^ Proteasomal processing compatibility was further assessed using the NetChop 3.0 server (C-terminal model, threshold 0.5) to predict cleavage sites along the vaccine sequence and verify that AAY and flanking regions were suitable for the generation of the intended CTL epitopes.

### Physicochemical properties analysis

The physicochemical evaluation was done to determine the molecular weight, Isoelectric point (pI) values, hydrophobicity, and stability of the vaccine constructs. Such evaluation was done using ExPASy ProtParam (https://www.expasy.org/resources/protpram), which provides the structural and functional properties of the vaccine construct.^22^ Moreover, solubility analysis was performed using SoluProt 1.0, which assessed the construct reliability toward further experimental validation.

### Tertiary structure prediction and validation

The vaccine construct's tertiary structure was predicted using AlphaFold3 (https://foldserver.com/, a deep-learning-based protein structure prediction tool developed by DeepMind.^23^ The program utilizes Deep Neural Networks (DNNs) in conjunction with Multiple Sequence Alignments (MSAs) to predict the atomic-level structure of proteins, thereby facilitating the development of vaccines and drugs. To validate the predicted models, the Ramachandran plot from the PROCHECK server (https://saves.mbi.ucla.edu/) was used to validate based on the stereochemical precision of the provided models.^24^

### Molecular docking and interaction analysis

The protein-protein docking for vaccine construct V2 with the TLR4 was performed with the ClusPro 2.0 server, available at (https://cluspro.bu.edu/publications.php). To analyze the binding interaction of the vaccine construct with the TLR4 receptor, the docked complex of the TLR4 vaccine was uploaded onto the PDBsum server (https://www.ebi.ac.uk/thornton-srv/databases/pdbsum/Generate.html). This tool provided interaction mapping that showed hydrogen bonds, salt linkages, disulfide bonds, and non-covalent contacts. Residue counts at the interface, interface Area (Å²), and some participating amino acids were also revealed, indicating the informativeness of residue-level interactions within the vaccine-receptor complex.^25^

### Molecular dynamics simulations

To assess the dynamic behavior and structural stability of the vaccine-TLR4 complex, MD simulations were conducted using the Desmond (D.E. Shaw Research). Before running the simulation, the complex was first refined with the Protein Preparation Wizard in Maestro, which included both optimization and energy minimization. The system setup was performed using the System Builder tool, where in each complex was solvated in an orthogonal box with Simple Point Charge (SPC) water molecules placed at a distance of 10 Å from the box edges. The solution was neutralized with Na⁺ and Cl⁻ ions, and 0.15 M NaCl was introduced to simulate physiological conditions. The energy minimization procedure was completed under the NPT ensemble. Each production simulation was performed throughout 100 ns at 300 K and 1 atm, applying the OPLS4 force field. This was done after all models had undergone equilibration. Electrostatic short-range interactions were calculated with the PME method and a 9.0 Å cutoff for Coulomb interactions. The pressure was controlled using the Martyna-Tuckerman-Klein chain barostat, and the temperature was controlled with the Nosé-Hoover thermostat, which had a coupling constant of 2.0 ps. Data was saved every 100 ps during the simulation. Conformational and structural changes were assessed using RMSD and RMSF analysis. Furthermore, the Radius of gyration (Rg) was calculated to measure compactness over time. The trajectories from Desmond were converted into XTC format with MDTraj, and Rg was calculated with the gmx gyrate command in GROMACS.^26^

### Principal component analysis

The Principal Component Analysis (PCA) was performed using the C-alpha atomic coordinates from the MD simulation trajectories. Each resulting atomic position contains a covariance matrix formed for independent diagonals, resulting in a set of eigenvectors and eigenvalues, where energy represents the principal components of motion, or PCs. The dominant motions were examined by projecting the coordinates into the Geometric Algorithms rotary owning these PCs. This analysis was performed using GROMACS with trajectory files in XTC format. The generation of the covariance matrix and its diagonalization was performed in the gmx covar, and atomic displacements with respect to the principal components were performed with the gmx anaeig with the proj flag.^27^

### Dynamic cross-correlation matrix

The Dynamic Cross-Correlation Matrix (DCCM) was used to study the correlated and anti-correlated movements of residues during molecular dynamics simulations. It allows analyzing the movement patterns throughout the entire protein by assessing the movement of residue pairs over time. For this study, the movements of the Cα atoms were considered as a proxy for the dynamics of the residue level. The cross-correlation coefficient between the residues i and j, C (i, j), is computed as follows:

$$C\;\left(i,j\right)=\frac{\langle \Delta r_{i}\;.\;\Delta r_{j}\;\rangle }{<{\left|\Delta r_{i}\right|}^{2}\;{>}^{\frac{1}{2}}\;<\;{\left|\Delta r_{j}\right|}^{2}{>}^{\frac{1}{2}}}$$

Where, Δrᵢ is the displacement vector of the C-alpha atom with respect to the mean position. The correlation coefficient C (i, j)>0 shows positive correlation, C (i, j)>0 indicates negative correlation (anti-correlation), whereas C (i, j) = 0 indicates no correlation.

MDTraj was used first to convert DCD to XTC format using trajectories generated in the Desmond module, subsequently performing DCCM calculation using R language from the Bio3D package.^28^

#### Free energy landscape

The Free Energy Landscape (FEL) analysis gives information on the energy differences of the protein conformations that are sampled in the course of Molecular Dynamics (MD) simulations by evaluating changes along the principal components derived from PCA. This helps in the detection of metastable states and their corresponding free energies. For this study, FEL analysis was carried out using GROMACS. Gibbs free energy was evaluated as a function of the first two principal components, PC1 and PC2. Using the gmx sham tool, the free energy profiles were determined. To better visualize the data, Locally Estimated Scatterplot Smoothing (LOESS) regression was used to smooth the FEL plots. Furthermore, Kernel Density Estimation (KDE) plots based on PCA results were used to validate the FEL distributions, ensuring consistency in conformational sampling.

#### In silico immune simulation analysis

To evaluate the immunogenic potential of the designed vaccine construct, an in silico immune simulation was conducted using the C-ImmSim server (https://kraken.iac.rm.cnr.it/C-IMMSIM/index.php). The simulation parameters were set with a random seed value of 12,345, a simulation volume of 10, and a total of 100-time steps. A single injection of the vaccine was administered at time step 1, simulating a primary immune response. The vaccine was introduced without the presence of lipopolysaccharide to mimic a non-endotoxic formulation. An adjuvant strength of 100 was included to enhance the immune response, along with the injection of 1000 antigenic units. Under these conditions, all basic parameters of adaptive and non-specific response evaluation, including lymphocytes and the production of cytokines and cells that provide immunological memory, were achieved.^29^

#### In silico cloning

The initial step in cloning involves converting the vaccine sequence from its amino acid form into a nucleotide sequence, which was performed using EMBOSS Backtranseq (https://www.ebi.ac.uk/jdispatcher/st/emboss_transeq). This was followed by codon optimization of the vaccine construct using ExpOptimizer from the NovoPro server (https://www.ebi.ac.uk/jdispatcher/st/emboss_transeq), which was done to maximize gene expression in the designated host organism. SnapGene v8.0.3 software was utilized for insertion and cloning. This program enables accurate plasmid construction, selection of appropriate restriction enzymes for cloning, and in silico cloning, which streamlines the efficiency and precision of the actual cloning process for later experimental use.^30^

This study did not involve human or animal subjects and therefore did not require adherence to CONSORT, STROBE, ARRIVE, or PRISMA guidelines. Instead, all analyses followed established computational immunoinformatics pipelines.

## Results

### Antigenicity and structural features of protein P28729

The primary sequence of the Envelopment glycoprotein of the Seoul virus was retrieved from UniProt with the UniProt ID of P28729. The protein was found to be antigenic, with a score of 0.5489, as predicted by the VaxiJen v2.0 server. Furthermore, the TMHMM analysis of the Envelopment glycoprotein (P28729) predicts three distinct transmembrane helices. These helices are located between residues 485‒507, 624‒646, and 1106‒1128. Both the N-terminal and C-terminal regions of the protein are predicted to be located between the transmembrane helices. Thus, the expected topology aligns with the possibility of P28729 being involved in signaling or transport activities essential to its biological function.

### Evolutionary conservation of the envelope glycoprotein

**Fig. 1:**
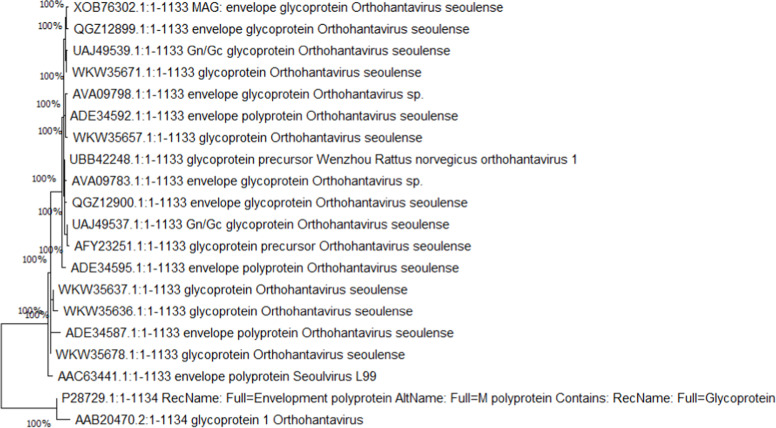
Neighbor-Joining phylogenetic tree of Seoul virus envelopment glycoprotein sequences. Bootstrap values (%, 1000 replicates) are shown at the nodes; 100% denotes full support. The scale bar represents amino-acid substitutions per site. The study sequence involves Uniprot P28729. Clade colors correspond to the taxa/groups shown in the legend.

The envelope glycoprotein of the Seoul virus was subjected to an evolutionary analysis by performing a BLASTp search for homologous proteins, constructing a phylogenetic tree in MEGA11 using the Neighbor-Joining method. The resultant tree suggested that the glycoprotein of interest has a close similarity with other strains of the Ortho Hantavirus sequence due to high sequence similarity. Bootstrap values (percentage from 1000 replicates) are shown at the nodes, with 100% indicating full support. The study sequence has UniProt P28729 in the tree (Fig. 1). Interestingly, some sequences from more distantly related strains, such as Wenzhou *R. norvegicus Ortho hantavirus* and orthohantavirus sp., were found in close association, indicating evolutionary diversification of these but closely related viruses. This confirms the evolutionary relationship and conservation of the selected protein, thereby strengthening its selection as a reliable antigenic target for the development of a broad-spectrum vaccine against the Seoul virus.

### Predicted MHC-I and MHC-II epitopes

Using this screening strategy, 16 epitopes for MHC-I (Table 1) and 16 epitopes for MHC-II (Table 2) were selected for vaccine design. These epitopes demonstrated strong immunogenic potential, substantial binding affinity, and favorable safety profiles, supporting their suitability as core components of a multi-epitope vaccine. Sixteen epitopes per class were selected to ensure broad immune coverage across genetically diverse human populations with heterogeneous HLA allele distributions, while maintaining a structurally feasible construct. This number represents an optimal balance between maximizing population coverage and avoiding excessive construct length that could compromise protein stability or expression efficiency. This epitope count is consistent with the size of epitope pools reported in other experimentally validated multi-epitope vaccine constructs, where a comparable number of epitopes has been shown to achieve broad HLA coverage while maintaining construct feasibility and structural integrity. All selected epitopes exhibited high antigenicity scores, indicating strong potential to elicit protective immune responses. To minimize epitope redundancy and reduce the risk of immunodominance or epitope suppression, overlapping or redundant epitopes were systematically excluded through a multi-step filtering strategy. Epitope prioritization was guided by antigenicity, HLA allele coverage, non-toxicity, and non-allergenicity, with preference given to unique epitopes that maximized immune breadth. All retained epitopes were further validated as non-allergenic using AllerTOP, ensuring their independent immunological contribution and safety within the final multi-epitope vaccine construct.

**Table 1:** Predicted MHC class I binding epitopes from the selected antigenic proteins. For each epitope, antigenicity score, allergenicity, and toxicity are provided.

Protein	Epitopes	Antigenicity	Allergenicity	Toxicity	IC₅₀ Rank
Envelopment glycoprotein	FRIISVRYSR	1.0068	Non-allergen	Non-Toxic	9.37
	GLVPYRIQV	1.5169	Non-allergen	Non-Toxic	35.92
	LTKVVWRKK	0.8003	Non-allergen	Non-Toxic	9.88
	RNTYELDFSF	1.8687	Non-allergen	Non-Toxic	37.38
	IAFAGIPSY	0.8163	Non-allergen	Non-Toxic	6.84
	TLAILLVLK	1.0401	Non-allergen	Non-Toxic	22.37
	VSFKGLCMLK	1.7242	Non-allergen	Non-Toxic	12.31
	LLITFCFGWV	1.1024	Non-allergen	Non-Toxic	13.52
	TICFFIHQK	1.4784	Non-allergen	Non-Toxic	5.27
	RYSRKVCVQF	1.059	Non-allergen	Non-Toxic	19.33
	TETATQAHYK	0.8993	Non-allergen	Non-Toxic	17.6
	LVPYRIQVVY	1.1141	Non-allergen	Non-Toxic	63.8
	QQISFICQR	2.3992	Non-allergen	Non-Toxic	34.47
	RIISVRYSRK	0.9979	Non-allergen	Non-Toxic	16.93
	CTLAILLVLK	0.7691	Non-allergen	Non-Toxic	34.01
	IISVRYSRK	2.0063	Non-allergen	Non-Toxic	39.97

**Table 2:** Predicted MHC class II binding epitopes from the selected Envelopment glycoprotein. Antigenicity, allergenicity, and toxicity are listed for each epitope.

Protein	Epitopes	Antigenicity	Allergenicity	Toxicity	IC₅₀ Rank
Envelopment glycoprotein	CFVPDKAVVSALKRG	0.8504	Non-allergen	Non-Toxic	6.7
	DMAICYGAESVTLSR	0.922	Non-allergen	Non-Toxic	39.7
	IISVRYSRKVCVQFG	1.9177	Non-allergen	Non-Toxic	51.7
	KNLKLIAFAGIPSYS	0.8388	Non-allergen	Non-Toxic	25.8
	LIGLVPYRIQVVYER	1.2383	Non-allergen	Non-Toxic	26.2
	NLKLIAFAGIPSYSS	0.8469	Non-allergen	Non-Toxic	22.9
	PFRIISVRYSRKVCV	1.0723	Non-allergen	Non-Toxic	11.5
	TPFRIISVRYSRKVC	1.0215	Non-allergen	Non-Toxic	7
	PVATPFRIISVRYSR	0.7202	Non-allergen	Non-Toxic	11.2
	SSKNLKLIAFAGIPS	0.89	Non-allergen	Non-Toxic	32.1
	AALLITFCFGWVLIP	0.8646	Non-allergen	Non-Toxic	42.3
	AWGSGVGFTLTCQVS	0.7864	Non-allergen	Non-Toxic	53.8
	LYRTLNLFRYKSRCY	0.9465	Non-allergen	Non-Toxic	14
	CETLKELKAHNLSCV	0.8966	Non-allergen	Non-Toxic	19.1
	MAICYGAESVTLSRG	0.8555	Non-allergen	Non-Toxic	33.1
	GIFNITSPMCLVSKQ	0.8027	Non-allergen	Non-Toxic	39.2

### Assemblage of multi-epitope vaccine constructs

**Fig. 2:**
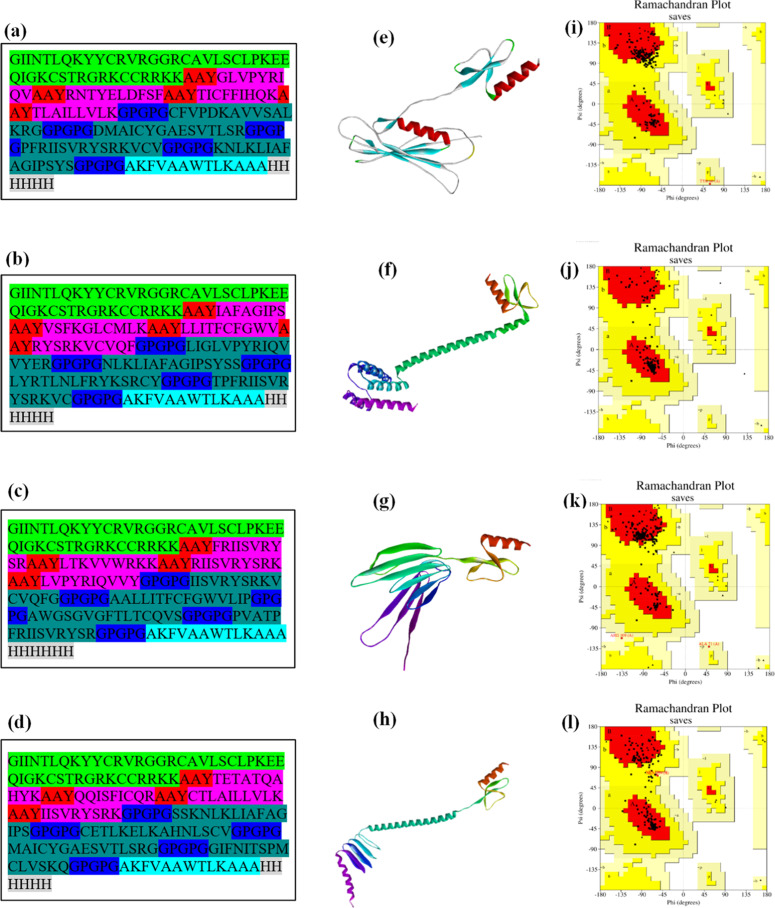
Linear sequences of vaccine constructs (a) V1, (b) V2, (c) V3, and (d) V4. Predicted tertiary structures of vaccine constructs (e) V1, (f) V2, (g) V3, and (h) V4 generated by AlphaFold3. In these panels, protein regions are color-coded according to AlphaFold3 pLDDT confidence scores: blue (> 90, very high), cyan (70–90, high), yellow (50–70, low), and orange/red (< 50, very low). (i) PROCHECK Ramachandran plots of predicted tertiary structures for V1, (j) V2, (k) V3, and (l) V4. Background colors indicate standard PROCHECK regions: Red = Most favored, Yellow = Additionally allowed, Light yellow = Generously allowed, and White = Disallowed regions. Percentages of residues in most favored regions: V1 = 74.0%, V2 = 95.6%, V3 = 83.0%, V4 = 74.1%. The axes represent φ (x-axis) and ψ (y-axis) backbone dihedral angles.

The selected epitopes of MHC-I and MHC-II, with high antigenic scores and non-allergenic capabilities, were used for vaccine design. To enhance the spacing of these epitopes and, thus, their immunogenicity, AAY and GPGPG linkers were used to join them all together. β-defensin-3 adjuvant was placed at the vaccine C-terminus to boost the immune response, while PADRE was added at the N-terminus, and a 6X histidine tag for purification. As shown in Fig. 2 (a, b, c, & d), a total of four different constructs were designed using various combinations of epitopes, and four multi-epitope vaccine constructs (V1–V4) were generated using the same adjuvant, PADRE sequence, and linker strategy, but differed in epitope composition and arrangement. V1 was enriched with epitopes showing the highest predicted antigenicity and strong binding across multiple HLA alleles. V2 was designed as a balanced construct that maximized global and regional HLA population coverage while maintaining favorable physicochemical properties and avoiding clustering of epitopes with similar HLA specificities. V3 and V4 incorporated alternative epitope subsets to explore different combinations of antigenicity, stability, and population coverage. A detailed comparison of the four constructs in terms of MHC-I and MHC-II epitope composition, predicted antigenicity, and HLA population coverage is provided in Supplemental Table S1.

Proteasomal cleavage prediction using NetChop (threshold 0.5) identified 69 potential cleavage sites in the 199-amino-acid V1 construct, 71 sites in V2, 73 sites in V3, and 61 sites in V4. The majority of high-score cleavage signals were located within or adjacent to the AAY and GPGPG linker regions, with only a limited number falling within epitope cores. This pattern indicates that the designed junctions are broadly compatible with proteasomal processing and release of the intended epitopes, while the few predicted internal sites should be interpreted as probabilistic and not necessarily disruptive to epitope generation.

### Global population coverage of vaccine constructs

**Fig. 3:**
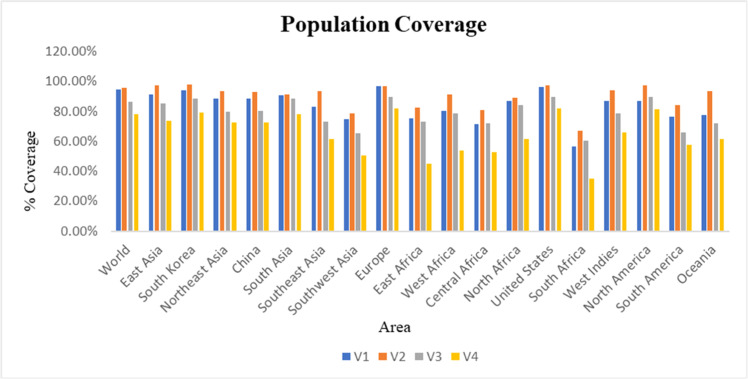
Population coverage of alleles for selected epitope combinations in vaccine construct. Blue Bars represent V1, Orange Bars represent V2, Grey Bars represent V3, and Yellow Bars represent V4.

Using the IEDB Population Coverage Analysis Tool, four multiepitope vaccine designs, V1, V2, V3, and V4, were evaluated for global and regional population coverage based on HLA allele distribution. For each construct, the population coverage (%) corresponds to the proportion of individuals in a given region predicted to present at least one of the epitopes included in that vaccine construct, based on regional HLA allele frequency data from the IEDB population coverage tool. V2 demonstrated the highest global coverage at 95.94%, followed closely by V1 at 94.76%, indicating strong potential for worldwide applicability. Both constructs also showed exceptionally high coverage in Europe (96.98% for V2 and 97.07% for V1), South Korea (97.82% for V2 and 94.15% for V1), and the United States (97.22% for V2 and 96.15% for V1). V3 achieved 86.30% global coverage, and V4 had 78.33%. In contrast, V1 and V2 showed notable strengths in China (93.06% for V2 and 88.43% for V1), as well as in other high-burden areas documented in the Introduction. These results suggest that V1 and V2, in particular, are well-suited to provide broad protection in both global and region-specific contexts (Fig. 3).

### Physicochemical properties of vaccine constructs

Molecular weights range from 21.13 kDa (V4) to 21.89 kDa (V3), indicating manageable sizes for vaccine delivery. Instability index values confirm that all constructs are stable, with V4 being the most stable (31.70). The aliphatic index highlights thermostability, with V3 scoring the highest (82.40) and V4 the lowest (78.09). GRAVY scores are negative for all constructs, reflecting their hydrophilic nature, which supports better solubility and biological interactions. The physicochemical properties of all vaccine constructs are presented in Table 3. The instability index values confirm that all constructs are stable, with V4 being the most stable (31.70). The aliphatic index underscores thermal stability, with V3 scoring the highest (82.40), followed by V4 (78.09). The GRAVY scores were also negative for all constructs, demonstrating their hydrophilic nature, which supports better solubility and biological interactions.

**Table 3:** Physicochemical Properties of vaccine constructs V1, V2, V3, and V4.

Vaccine Constructs	Probable Non allergen	Probable Antigenic	Solubility	Molecular Weight	N° of Amino acids	Theoretical PI	Instability Analysis	Aliphatic Index	GRAVY
Server Used	AllerTOP	VaxiJen	SoluProt	ProtParam	ProtParam	ProtParam	ProtParam	ProtParam	ProtParam
V1	+	0.688	0.773	21,324.06	198	9.93	17.39	81.36	−0.020
V2	+	0.6830	0.635	21,822.80	199	10.16	16.17	79.95	−0.011
V3	+	0.5931	0.890	21,892.85	200	10.64	16.33	82.40	−0.017
V4	+	0.6879	0.812	21,126.74	199	9.79	31.70	78.09	−0.110

### Predicted tertiary structure of vaccine constructs

Using AlphaFold3, the tertiary structures of the four vaccine constructs, V1, V2, V3, and V4, were predicted, as depicted in Fig. 2 (e, f, g, & h). Protein regions are color-coded according to AlphaFold3 pLDDT confidence scores: blue indicates very high confidence (> 90%), cyan indicates high confidence (70%‒90%), yellow indicates low confidence (50%‒70%), and orange/red indicates very low confidence (< 50%). The predicted structures provide detailed insights into the spatial arrangement of amino acids, which is crucial for understanding the stability and potential immunogenicity of the constructs. The low-confidence yellow/orange regions correspond mainly to the flexible AAY and GPGPG linker segments and terminal regions, whereas the majority of CTL and HTL epitope cores and the β-defensin-3 adjuvant domain fall within medium- to high-confidence regions. This indicates that key epitopes are unlikely to be structurally compromised in the predicted models. These structural models facilitate the identification of conformational epitopes and aid in the rational design of vaccines by highlighting regions critical for immune recognition. However, given that the constructs are long artificial multi-epitope proteins, the predicted structures should not be interpreted as evidence of a single stable globular fold, and experimental studies will be required to verify their actual folding and stability. The high confidence in these predictions supports their utility in guiding subsequent experimental validations and vaccine development efforts.

### Validation of predicted tertiary structures of vaccine constructs V1, V2, V3 &V4

The tertiary structures of the four vaccine constructs (V1, V2, V3, and V4) were validated using PROCHECK Ramachandran plots, which assess the φ and ψ dihedral angles of amino acid residues. The background grid and color coding (most favored, additionally allowed, generously allowed, and disallowed regions) are standard for PROCHECK and remain consistent across panels; differences are evident in the residue distribution. V2 showed the highest percentage of residues in favored areas (95.6%), followed by V3 (83.0%), V1 (74.0%), and V4 (74.1%), indicating that V2 has the most stereochemically reliable structure (Fig. 2i, j, k, & l).

### Molecular docking and interaction analysis of the V2 vaccine construct with TLR4

The vaccine candidate V2 was analyzed for docking interactions with the Toll-like receptor TLR4 using the ClusPro 2.0 server. ClusPro produced 30 docking complexes for both receptor interactions. The docking results indicated that the interaction between V2 and TLR4 exhibited the lowest binding energy of −1382.2, with 70 interacting members, signifying a strong and stable interaction.

**Fig. 4:**
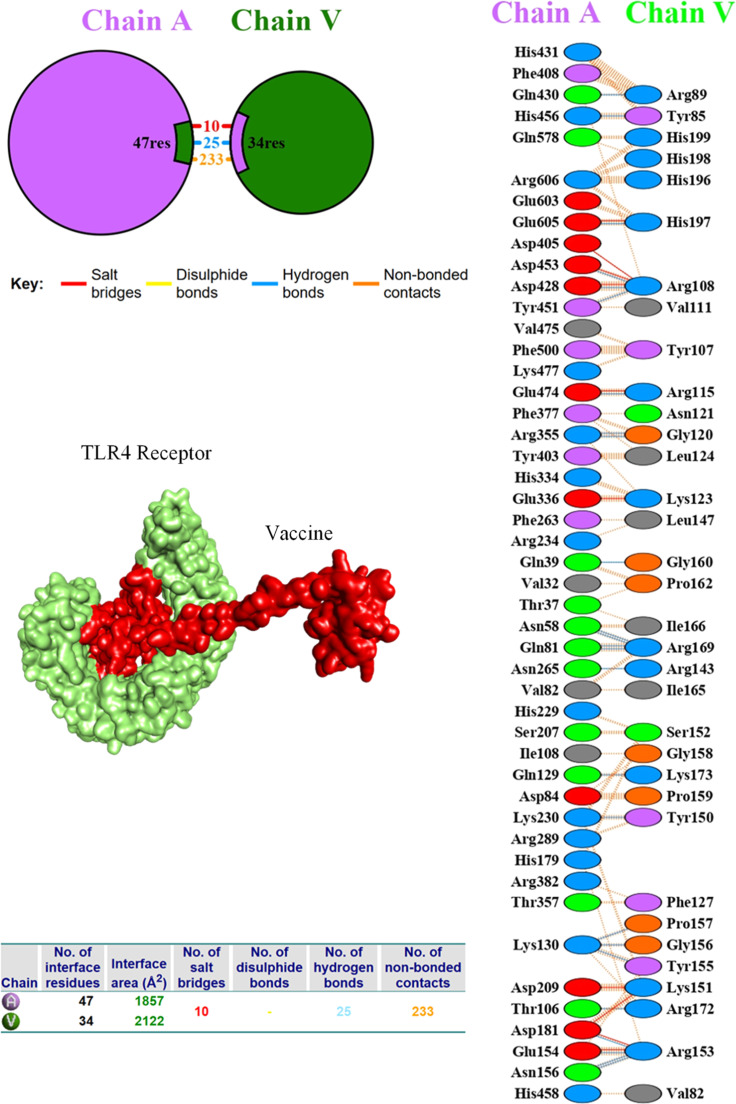
Interaction analysis between the designed vaccine (Chain V, green) and the human TLR4 receptor (Chain A, purple) performed using the PDBsum server. Blue dashed lines indicate hydrogen bonds, red lines represent salt bridges, and orange lines denote non-bonded interactions.

The interaction analysis between the vaccine construct, shown as chain V, and human TLR4 chain A, performed using the PDBsum server, is shown in Fig. 4. The interface comprised 81 residues including 25 hydrogen bonds, 10 salt bridges, and 233 non-bonded contacts. The interface contains 47 residues of TLR4 and 34 residues of the vaccine, corresponding to a total buried surface area of 1857 Å² (TLR4) and 2122 Å² (vaccine). The 2D schematic illustrates the critical residues that are crucial for interaction. Notable for TLR4 are residues ASN58, GLN81, THR106, GLU154, LYS130, and ASP181, which formed strong hydrogen bonds with the positively charged residues ARG169, ARG153, ARG172, and TYR155 from the vaccine construct. Of all the hydrogen bonds, the shortest ones, LYS130–GLY156 (2.54 Å) and GLN129-LYS173 (2.60 Å), suggest strong interactions and are likely to be critical for effective molecular recognition. Furthermore, the presence of 10 salt bridge electrostatic interactions, which are usually stronger than hydrogen bonds, adds greater stability to the complex. The presence of these salt bridges, alongside the hydrogen bonds, indicates a strong and precise interaction between the vaccine and the TLR4 receptor, which is crucial for triggering a robust immune response.

### Molecular dynamics simulation of vaccine construct V2-TLR4 complex

**Fig. 5:**
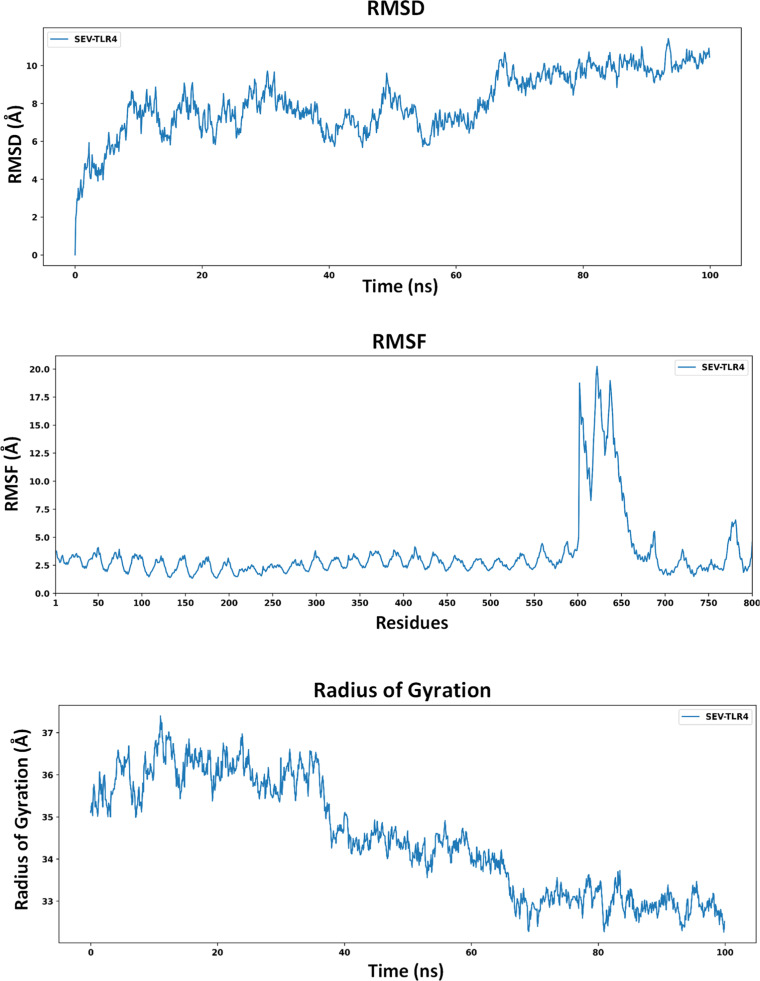
(a) RMSD graph of vaccine-TLR4 complex observed over 100 ns of MD simulation, (b) RMSF graph, (c) Radius of Gyration analysis of the vaccine-TLR4 complex.

The Root Mean Square Deviation (RMSD) was assessed to monitor the conformational behavior of the vaccine-TLR4 complex over a 100 ns MD simulation (Fig. 5a). At the start of the simulation, there was a sharp increase in RMSD during the first 10 ns, indicating initial conformational adjustments and relaxation of the complex within the simulation box. After 10 ns, the RMSD gradually increased from 6 Å to 8 Å, with a pronounced dip around 45‒55 ns, suggesting a transient structural rearrangement towards a more compact form. Following 60 ns, the RMSD values rose again and fluctuated between approximately 8 and 10 Å for the remainder of the simulation. The mean RMSD over 100 ns was 7.8 Å, consistent with a complex that exhibits noticeable structural flexibility while remaining associated throughout the simulation timeframe, rather than indicating rigid long-term stability.

The Root Mean Square Fluctuation (RMSF) was analyzed to evaluate residue-level mobility within the vaccine-TLR4 complex over the 100 ns simulation (Fig. 5b). RMSF values for most residues, particularly in the TLR4 region, remained within an approximate range of 2.0‒4.5 Å, reflecting moderate local fluctuations. In contrast, the C-terminal region corresponding to the vaccine construct displayed increased flexibility, with residues around frames 600–680 showing elevated RMSF values and a maximum of 19.2 Å. Such enhanced mobility is expected for peripheral terminal residues and flexible loops, which often lack strong secondary structural constraints in vaccine constructs. The average RMSF for the complex was 3.6 Å, suggesting overall dynamic but not excessive fluctuations. Within the 100 ns window, these observations indicate that the complex maintains its interaction while allowing flexible regions of the vaccine to sample conformations that could facilitate antigen exposure and immune recognition, although longer simulations would be required to draw conclusions about long-term stability.

The Radius of gyration (Rg) is useful for assessing the compactness of protein complexes during molecular dynamics simulations. The Rg of the vaccine-TLR4 complex was monitored over a 100 ns simulation (Fig. 5c). Within the first 20 ns, the maximum Rg value reached 37.2 Å, increasing from an initial value of 35.2 Å at 0 ns, suggesting a modest expansion associated with initial structural rearrangements and equilibration. After approximately 40 ns, Rg began to decrease gradually and, in the last 20 ns of the simulation, fluctuated around 32.8 Å. These changes indicate a tendency toward a more compact conformation within the simulated timeframe. The average Rg value over 100 ns was approximately 34.6 Å, consistent with a complex that explores different conformational states while retaining an overall compact organization during the simulation period, although longer simulations would be required to fully characterize long-term conformational stability.

### Principal component analysis

**Fig. 6:**
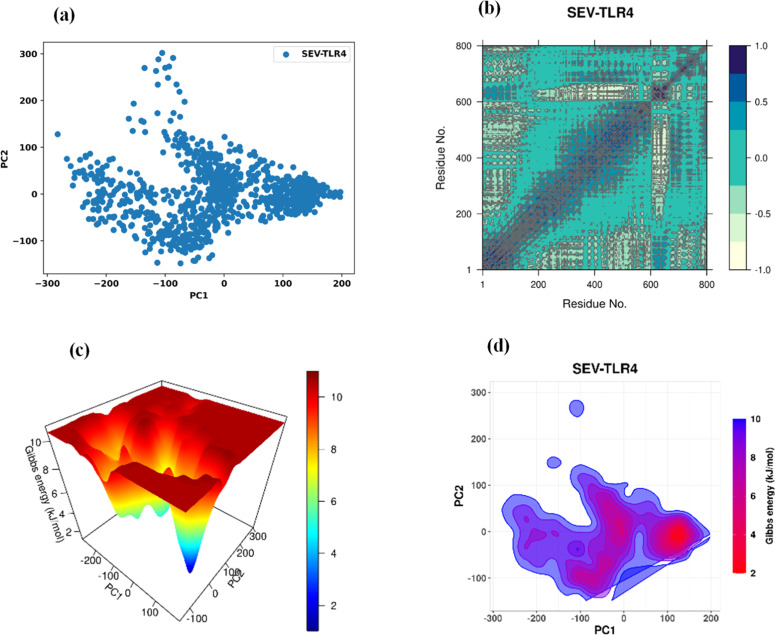
(a) PCA plot of vaccine-TLR4 complex, (b) Dynamic Cross-Correlation Matrix (DCCM), (c) Three-Dimensional Free Energy Landscape of the vaccine-TLR4 complex, (d) Two-Dimensional Free Energy Landscape (2D FEL Contour Plot).

Principal Component Analysis (PCA) was performed to characterize the dominant collective motions of the vaccine-TLR4 complex during the 100 ns molecular dynamics simulation. Fig. 6a shows the distribution of conformations projected onto the first two principal components (PC1 and PC2), which capture the main large-scale motions of the system. The trajectory is spread over a relatively broad region of PC space, with PC1 values ranging from approximately −250 to +200 and PC2 from −150 to +300, indicating that multiple conformational states are sampled, particularly in the more flexible regions of the multi-epitope vaccine construct. A denser clustering of points in the lower right quadrant suggests that the complex spends a substantial portion of the simulation in a preferred conformational basin or metastable state, while still sampling additional conformations around it within the 100 ns window.

### Dynamic cross-correlation matrix

The Dynamic Cross-Correlation Matrix (DCCM) was used to examine correlated and anti-correlated atomic motions in the vaccine-TLR4 complex over the course of the simulation (Fig. 6b). The correlation coefficients range from −1.0 to +1.0, with positive values (blue) indicating residues that tend to move in a correlated manner and negative values (light to dark yellow) indicating anti-correlated motions. The matrix reveals several regions, particularly within the approximate residue range 200–600, that exhibit noticeable positive correlations, consistent with coordinated motions among segments of the complex. Patches of weaker anti-correlated interactions are also observed, suggesting localized flexibility and opposing movements in specific regions. Together, these patterns reflect a balance of coordinated and flexible motions characteristic of a dynamically stable complex within the simulated timescale.

### Free energy landscape

To examine the conformational behavior of the vaccine-TLR4 complex, a principal component analysis-based Free Energy Landscape (FEL) analysis was performed. The 3D FEL plot (Fig. 6c) showed several energy basins, with the deepest minimum at approximately 2.0 kJ/moL, suggesting the presence of energetically favorable conformational states sampled during the simulation. The 2D contour FEL (Fig. 6d) displayed intense low-energy regions (2.0‒4.0 kJ/moL) surrounded by progressively higher-energy areas up to 10.0 kJ/moL, consistent with transitions between more and less favorable conformations within the simulated timescale. These results indicate that, over 100 ns, the vaccine-TLR4 complex predominantly occupies one or a few low-energy conformational basins while intermittently exploring higher-energy states, but they do not by themselves establish long-term conformational stability.

Although the vaccine-TLR4 complex remained associated and showed a tendency toward compaction during the 100 ns simulation, the mean RMSD value of 7.8 Å indicates appreciable conformational flexibility; therefore, stability in this context refers to sustained interaction without major unfolding rather than a rigid or static structure.

### *In silico* immune simulation of vaccine construct V2

**Fig. 7:**
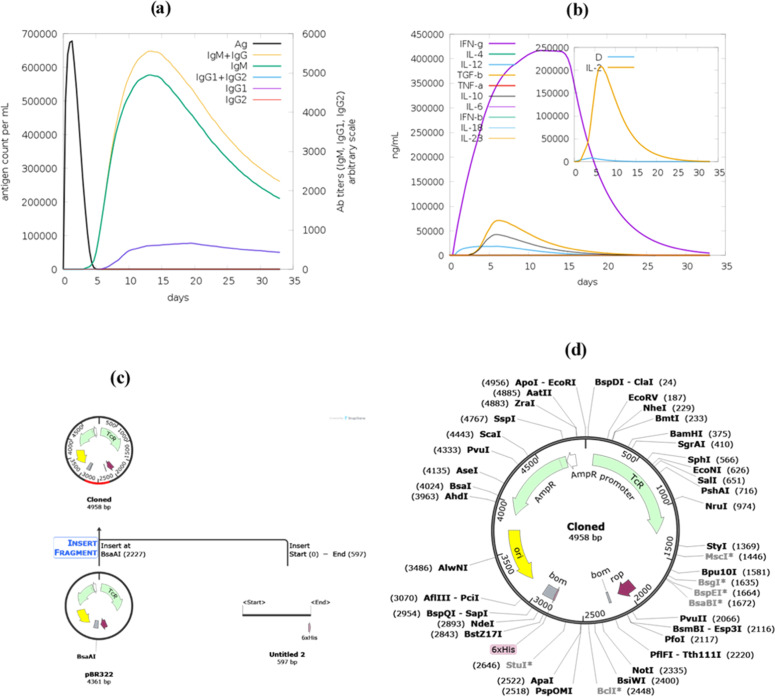
(a) Simulated antigen clearance and antibody production. (b) Cytokine profile following vaccination, showing dominant IFN-γ and IL-2 peaks, indicating a strong Th1 immune response. (c) Cloning history showing the insertion of V2 into pBR322 at the BsaA1 restriction site. (d) Cloned map of V2 in the pBR322 vector.

The *in silico* immune simulation of V2 conducted using C-ImmSim predicted an adaptive immune response pattern characterized by antigen clearance, antibody production, and memory cell formation. The antigen count peaked at approximately 650,000 antigens/mL on Day 5 and then declined over time, consistent with model-predicted immune-mediated clearance. The simulated antibody response began with IgM, which peaked at around 1000 on Day 10, followed by a class switch to IgG antibodies, with IgG1 reaching a peak of about 4500 on Day 15 and IgG2 around 2000 (Fig. 7a). Cytokine profiles in the simulation suggested a predominantly Th1-biased response, with IFN-γ peaking at approximately 450,000 ng/mL on Day 5, accompanied by early increases in IL-2 (50,000 ng/mL) and TNF-α (150,000 ng/mL), and a later rise in IL-10 (50,000 ng/mL on Day 15), while IL-4 remained comparatively low (10,000 ng/mL) (Fig. 7b). These findings should be interpreted as model-based, *in silico* predictions that support the potential of V2 to elicit a coordinated immune response and warrant further *in vitro* and *in vivo* validation, rather than as direct evidence of actual immune activation in humans.

### *In silico* cloning of vaccine construct V2

The V2 construct, consisting of 200 amino acids, was reverse translated into a nucleotide sequence (597 nucleotides) using EMBOSS Transeq. This initial reverse-translated sequence had a GC content of 67.67%, which is relatively high and not optimal for expression in *Escherichia coli*. The sequence was then codon-optimized using the ExPASy Optimizer to enhance expression in the host organism, reducing the GC content to 56.62%, a value more compatible with efficient transcription and mRNA stability in *E. coli*. After codon optimization, the vaccine sequence was inserted into the pBR322 vector for cloning using SnapGene software. The original size of the pBR322 vector was 4361 bp (Fig. 7c), and after insertion of the 597 bp vaccine fragment, the size of the recombinant plasmid increased to 4958 bp (Fig. 7d). This successful insertion indicates proper integration of the vaccine sequence into the plasmid, generating a construct suitable for subsequent experimental validation. Although pBR322 was used in this study for *in silico* cloning to demonstrate insertion and vector compatibility, modern expression systems such as pET-series vectors are more suitable for laboratory expression due to their strong promoters and capacity for high-level recombinant protein production.

## Discussion

This study presents a computational design of a multi-epitope vaccine against Seoul virus, a zoonotic pathogen causing Hemorrhagic Fever with Renal Syndrome, for which no approved vaccine or antiviral exists. Immunoinformatics approaches, including epitope mapping, structure prediction, molecular docking, immune simulation, and *in silico* cloning, were employed to develop the vaccine. Selected T-cell epitopes were chosen for high antigenicity, non-allergenicity, and non-toxicity to ensure robust humoral and cellular immunity. CTL epitopes were separated using AAY linkers to facilitate optimal MHC-I presentation, while HTL epitopes were joined with GPGPG linkers to enhance immune response and prevent epitope interference.

To enhance the innate immune response, a β-defensin-3 adjuvant was added to the C-terminus of the vaccine construct. β-defensin-3 is a small antimicrobial peptide with potent immunomodulatory properties. It can activate Antigen-Presenting Cells (APCs), such as dendritic cells and macrophages, enhancing innate immune responses. β-defensin-3 also recruits immune cells to the site of antigen presentation and promotes T-cell activation.^31^ In the context of recombinant multi-epitope vaccines, β-defensin-3 functions as a molecular adjuvant capable of enhancing antigen uptake and T-cell activation. PADRE (Pan HLA-DR-binding Epitope) was incorporated at the N-terminus of the vaccine to provide broad HLA coverage across diverse human populations. Its inclusion enhances CD4⁺ T-cell activation, which is critical for orchestrating immune responses, including supporting B-cell antibody production and assisting CD8⁺ T-cell cytotoxic functions. By facilitating stronger T-helper responses, PADRE contributes to the overall immunogenicity and long-term effectiveness of the vaccine.^32^

Based on the selected epitopes from MHC-I and MHC-II, four distinct vaccine constructs (V1, V2, V3, and V4) were designed. These constructs were evaluated for antigenicity, allergenicity, toxicity, solubility, molecular weight, and stability. AlphaFold3 was used for structure prediction, and the Ramachandran plot validated the structural integrity of the constructs. Among them, V2 proved the most promising, achieving the highest global population coverage of 95.94%, strong predicted antigenicity, and robust structural stability. High regional coverage in SEOV-endemic areas, South Korea (97.82%), China (93.06%), the USA (97.22%), and Europe (96.98%) supports its potential for broad protection, making V2 the lead candidate for further analysis. From a practical perspective, however, the expression and purification of a chimeric, epitope-rich protein of this length may be challenged by issues such as solubility, aggregation, and proteolytic degradation; although the *in-silico* analyses predict acceptable physicochemical properties, efficient production will require empirical optimization of the expression host and vector, possible use of solubility-enhancing fusion tags, and refinement of purification conditions.

An important consideration in multi-epitope vaccine design is the risk of epitope suppression or immunodominance, where only a limited subset of epitopes is preferentially recognized, reducing the effective breadth of the immune response. This risk was explicitly addressed in this study by selecting non-overlapping, non-redundant epitopes, separating them with AAY and GPGPG linkers, and evaluating multiple construct designs (V1–V4) to ensure balanced immunogenicity and broad HLA coverage. Experimental validation is required to confirm that all epitopes contribute effectively to the immune response. Docking analysis of V2 with human TLR4 showed strong interaction, with a binding energy of −1382.2, 25 hydrogen bonds, and 10 salt bridges. This indicates that the vaccine strongly engages innate immune receptors, which are crucial for antigen presentation and adaptive immunity activation. TLR4 recognizes viral components or harm signals and initiates immune responses via MyD88 and TRIF adaptors, leading to the expression of pro-inflammatory cytokines (*e.g.*, TNF-α, IL-6) and type I Interferons (IFN-α/β).^33^ Molecular dynamics simulations supported the docking results, showing that the V2–TLR4 complex maintained stable interactions over a 100 ns period. RMSD, RMSF, and radius of gyration values indicated a dynamically associated complex tending toward compact, receptor-compatible conformations. Principal component and free energy landscape analyses revealed that the complex predominantly sampled a limited number of low-energy states, while dynamic cross-correlation analysis showed coordinated residue motions indicative of functional flexibility at the interface. However, 100 ns is insufficient to confirm long-term stability, so these findings reflect behavior during the simulated window and highlight the need for longer simulations and experimental validation.

C-ImmSim predictions suggested that the vaccine could elicit a coordinated adaptive immune response, with antigen load peaks followed by IgM production and a class switch to IgG. Memory B- and T-cells were generated, and the cytokine profile indicated a predominantly Th1-biased response, relevant for intracellular pathogens like hantaviruses. These results, however, are *in silico* predictions and should be interpreted as hypotheses to guide experimental validation rather than direct evidence of *in vivo* immune activation. Careful selection of CTL, HTL, and B-cell epitopes ensured a broad immune response. CTL epitopes were predicted to bind multiple high-frequency HLA class I alleles, facilitating MHC-I presentation and CD8⁺ T-cell activation, essential for eliminating infected cells. HTL epitopes were chosen for high predicted binding to HLA class II alleles and IFN-γ induction, supporting CD4⁺ T-cell activation, B-cell maturation, and enhancement of CD8⁺ cytolytic activity. Collectively, this arrangement is expected to generate a balanced Th1/Th2 profile, promoting robust cellular immunity and durable antibody-mediated protection. Successful insertion into the pBR322 vector confirmed the construct’s feasibility for experimental validation and further development.

While there is currently no approved vaccine against SEOV, previous vaccine efforts against hantaviruses, including Hantaan Virus (HTNV), have largely focused on inactivated or attenuated whole-virus preparations, DNA vaccines, and viral-vectored or virus-like particle-based approaches.^34^ Compared to traditional hantavirus vaccine platforms, such as inactivated or live attenuated vaccines, DNA vaccines, and virus-like particle (VLP) or viral-vectored vaccines, the present study has proposed offers several advantages in terms of safety, immunogenicity, scalability, and production feasibility. Inactivated hantavirus vaccines, such as Hantavax for Hantaan Virus (HTNV), have been deployed and shown to induce neutralizing antibody responses, but issues such as waning antibody titers and limited long-term protection have been reported, indicating the need for improved efficacy.^35^ DNA vaccine strategies have been explored for hantaviruses, including HTNV and Sin Nombre Virus (SNV), demonstrating induction of neutralizing antibodies and protection in animal models, but their immunogenicity can be variable and delivery-dependent.^36^ Viral-vectored vaccines, such as Modified Vaccinia Ankara (MVA) vectors expressing hantavirus antigens, have shown promise in reducing viral loads and eliciting immune responses in preclinical studies.^37^ Similarly, VLP-based approaches engineered to present hantavirus antigens have been shown to elicit both humoral and cellular responses in animal models, leveraging particulate antigen presentation while avoiding the use of live virus.^38^ In contrast, the multi-epitope vaccine design targets a defined set of highly immunogenic epitopes, providing a more controlled immune response without the safety concerns associated with whole-virus vaccines and without reliance on complex vectors or particles.

Compared with these strategies, the present multi-epitope protein design offers a more defined antigenic composition, the ability to concentrate immune responses on conserved T- and B-cell epitopes, and the potential to reduce off-target or non-essential antigenic components; nonetheless, it also introduces practical uncertainties regarding expression yield, folding, and *in vivo* immunogenicity that can only be resolved empirically. In this sense, the construct proposed here should be viewed as a complementary, hypothesis-driven candidate that builds on, rather than replaces, prior SEOV vaccine approaches. Although the computational results are promising, the study faces challenges that necessitate further investigation. In general, this study makes a notable contribution to the formulation of a Seoul virus vaccine and demonstrates the efficacy of computational approaches in vaccine design aimed at responding to novel infectious disease outbreaks.

### Limitations

Although the results are promising, this study has several limitations. It assumes that the vaccine protein will fold correctly, that all epitopes will be properly exposed to the immune system, and that the linkers connecting the epitopes will function as intended *in vivo*. While *in silico* validations, including AlphaFold3 and Ramachandran analysis, support the folding stability, these predictions remain assumptions until experimentally confirmed. Similarly, computational predictions of immunogenicity and immune responses provide valuable insights but must be confirmed through *in vitro* and *in vivo* validation to ensure efficacy and safety. Furthermore, the vaccine construct targets TLR4 for immune activation, which may enhance immunogenicity; however, excessive stimulation of TLR4 could potentially trigger an overactive inflammatory response. Additionally, while the *in-silico* cloning into a pBR322 vector demonstrates successful integration, the actual expression of the vaccine in a host system such as *E. coli* may encounter challenges related to solubility, aggregation, and degradation. Therefore, experimental evaluation is essential to ensure both the safety and balanced immune activation, and successful cloning and expression of the vaccine.

## Conclusion

The study successfully designed and evaluated multiple multi-epitope vaccine constructs targeting the Seoul virus through a comprehensive immunoinformatics-based approach. Of all the constructs, V2 exhibited favorable physicochemical traits, high immunogenic potency, and significant coverage of the global population, thereby marking it as a promising candidate for further development. These findings provide the necessary groundwork for research on the SEOV vaccine. Further research should focus on validating these *in silico* predictions through *in vitro* and *in vivo* experiments, refining the vaccine design, and examining the protective efficacy of the constructs in preclinical/clinical trials. This work comprehensively addresses the sophisticated health risks surrounding the need to prevent SEOV infection and the health risks involved, and aids in the formulation of effective immunotherapeutic strategies.

## Ethical approval

Not applicable.

This study did not involve human participants, animal experiments, or clinical samples; hence, informed consent and ethical approval were not applicable.

## Availability of data and materials

All data generated and analyzed during this study are included in this published article. Additional raw data of this study are available at https://drive.google.com/drive/folders/1WnPNyJLTqJ3ubIr09mb44IvHkS4_WI5F?usp=drive_lin.

## Authors' contributions

Conceptualization, Muhammad Naveed; Methodology, Hafiz Muzzammel Rehman; Software, Muhammad Asim; Validation, Wafa Abdullah I. Al-Megrin and Ashwag Shami; Formal analysis, Maher S. Alwethaynani; Investigation, Abdullah A. Alqasem; Resources, Tariq Aziz; Data curation, Mohammed F. Abuzinadah; Writing-original draft preparation, Maher S Alwethaynani; Writing-review and editing, Ahmed M. Abdulfattah; Visualization, Majid Alhomrani; Supervision, Tariq Aziz and Muhammad Naveed.; Project administration, Muhammad Naveed; Funding acquisition, Tariq Aziz.

## Funding

The authors are thankful to Princess Nourah bint Abdulrahman University Researchers Supporting Project number (PNURSP2026R39), Princess Nourah bint Abdulrahman University, Riyadh, Saudi Arabia.

## Data availability

All data supporting the findings of this study are included within the manuscript. Additional raw data are available from the corresponding author upon reasonable request.

## Conflicts of interest

The authors declare no conflicts of interest.

## Appendix Supplementary materials

Image, application 1
