# Supplementary material for: Immunoinformatics-driven design of a multi-epitope vaccine against Seoul Virus: Structural, dynamic, and immunogenic profiling
Source: Clinics (Sao Paulo). 2026 May 12;81:100991. doi: 10.1016/j.clinsp.2026.100991 (PMC13195357; doi:10.1016/j.clinsp.2026.100991)
Supplement: Supplementary file 1 [file mmc1.docx]

**CLINICS-D-25-01089_Supplementary Material**

**Supplemental Table S1** Comparison of the four multi-epitope vaccine constructs (V1–V4) showing MHC-I and MHC-II epitope composition, predicted antigenicity of the constructs, and combined HLA population coverage (%).

| **Vaccine Construct** | **MHC-I Epitopes** | **MHC-II Epitopes** | **Antigenicity of Constrcut** | **Combined** **HLA Population Coverage (%) across World** |
| --- | --- | --- | --- | --- |
| V1 | GLVPYRIQV; RNTYELDFSF; TICFFIHQK; TLAILLVLK | CFVPDKAVVSALKRG; DMAICYGAESVTLSR; PFRIISVRYSRKVCV; KNLKLIAFAGIPSYS | 0.688 | 94.76 |
| V2 | IAFAGIPSY; VSFKGLCMLK; LLITFCFGWV; RYSRKVCVQF | LIGLVPYRIQVVYER; NLKLIAFAGIPSYSS; LYRTLNLFRYKSRCY; TPFRIISVRYSRKVC | 0.683 | 95.94 |
| V3 | FRIISVRYSR; LTKVVWRKK; RIISVRYSRK; LVPYRIQVVY | IISVRYSRKVCVQFG; AALLITFCFGWVLIP; AWGSGVGFTLTCQVS; PVATPFRIISVRYSR | 0.593 | 86.30 |
| V4 | TETATQAHYK; QQISFICQR; CTLAILLVLK; IISVRYSRK | SSKNLKLIAFAGIPS; CETLKELKAHNLSCV; MAICYGAESVTLSRG; GIFNITSPMCLVSKQ | 0.688 | 78.33 |
